# Primary Peritoneal Carcinomatosis: A Case Report

**DOI:** 10.7759/cureus.100774

**Published:** 2026-01-04

**Authors:** Anvitha Soundararajan, Rodrigo Furlan Silva Fabri, Barney Soskin

**Affiliations:** 1 Internal Medicine, Texas Tech University Health Sciences Center El Paso, Paul L. Foster School of Medicine, El Paso, USA

**Keywords:** adenocarcinoma, ascites, immunohistochemistry, müllerian carcinoma, ovarian cancer mimic, peritoneal carcinomatosis, primary peritoneal carcinoma

## Abstract

Primary peritoneal adenocarcinoma (PPC) is an uncommon malignancy that arises from the peritoneal lining and shares striking clinical, histopathological, and immunophenotypic similarities with epithelial ovarian carcinoma (EOC). Because both entities derive from Müllerian epithelium, distinguishing PPC from metastatic ovarian or gastrointestinal carcinoma poses a significant diagnostic challenge, particularly in the absence of detectable ovarian masses. Recognizing this rare entity is clinically relevant, as accurate classification influences prognosis, genetic counseling, and management strategies.

## Introduction

Primary peritoneal carcinoma (PPC) is a rare malignant neoplasm arising from the peritoneal lining that exhibits Müllerian epithelial differentiation [1]. It shares striking clinical, radiologic, and histopathologic similarities with epithelial ovarian carcinoma (EOC), often presenting a diagnostic dilemma in the absence of detectable ovarian masses [2,3]. Because these malignancies originate from the same embryologic tissue, their overlapping morphological features may delay accurate diagnosis and appropriate management [4].

Although uncommon, PPC predominantly affects postmenopausal women and typically presents with nonspecific symptoms such as abdominal distension, ascites, or dyspnea related to pleural effusion [1,5]. Imaging frequently demonstrates diffuse peritoneal thickening, omental caking, or serosal implants without significant ovarian enlargement, contributing to difficulty in distinguishing PPC from metastatic disease originating from ovarian, gastrointestinal, or pancreatic primaries [3,6].

Diagnosis requires correlation of imaging, cytology, and immunohistochemistry. Tumor cells in PPC characteristically express CK7, PAX8, and CA-125 while lacking CK20 expression, supporting Müllerian origin and excluding gastrointestinal sources [7,8]. Establishing this diagnosis is essential, as management parallels EOC treatment pathways, typically involving cytoreductive surgery followed by platinum-based chemotherapy [2,4].

We report the case of a 68-year-old female presenting with progressive dyspnea, pleural effusion, and diffuse peritoneal nodularity, ultimately diagnosed with primary peritoneal adenocarcinoma. This case highlights the key diagnostic challenges of PPC and underscores the importance of a multidisciplinary evaluation, integrating imaging, cytopathology, and immunohistochemistry, to distinguish it from metastatic disease and facilitate timely oncologic management.

## Case presentation

A 68-year-old Hispanic female with a past medical history of asthma presented to the emergency department with worsening shortness of breath for two days and progressive abdominal distension for one month. She also reported decreased appetite and unintentional weight loss of approximately 15 pounds. She denied fever, cough, or chest pain.

On physical examination, the patient appeared cachectic and mildly tachypneic. Vital signs revealed hypoxia with oxygen saturation of 88% on room air. The abdomen was distended with a fluid wave, and bilateral lower-extremity edema was noted. Breath sounds were decreased bilaterally, more pronounced on the right side.

**Figure 1:**
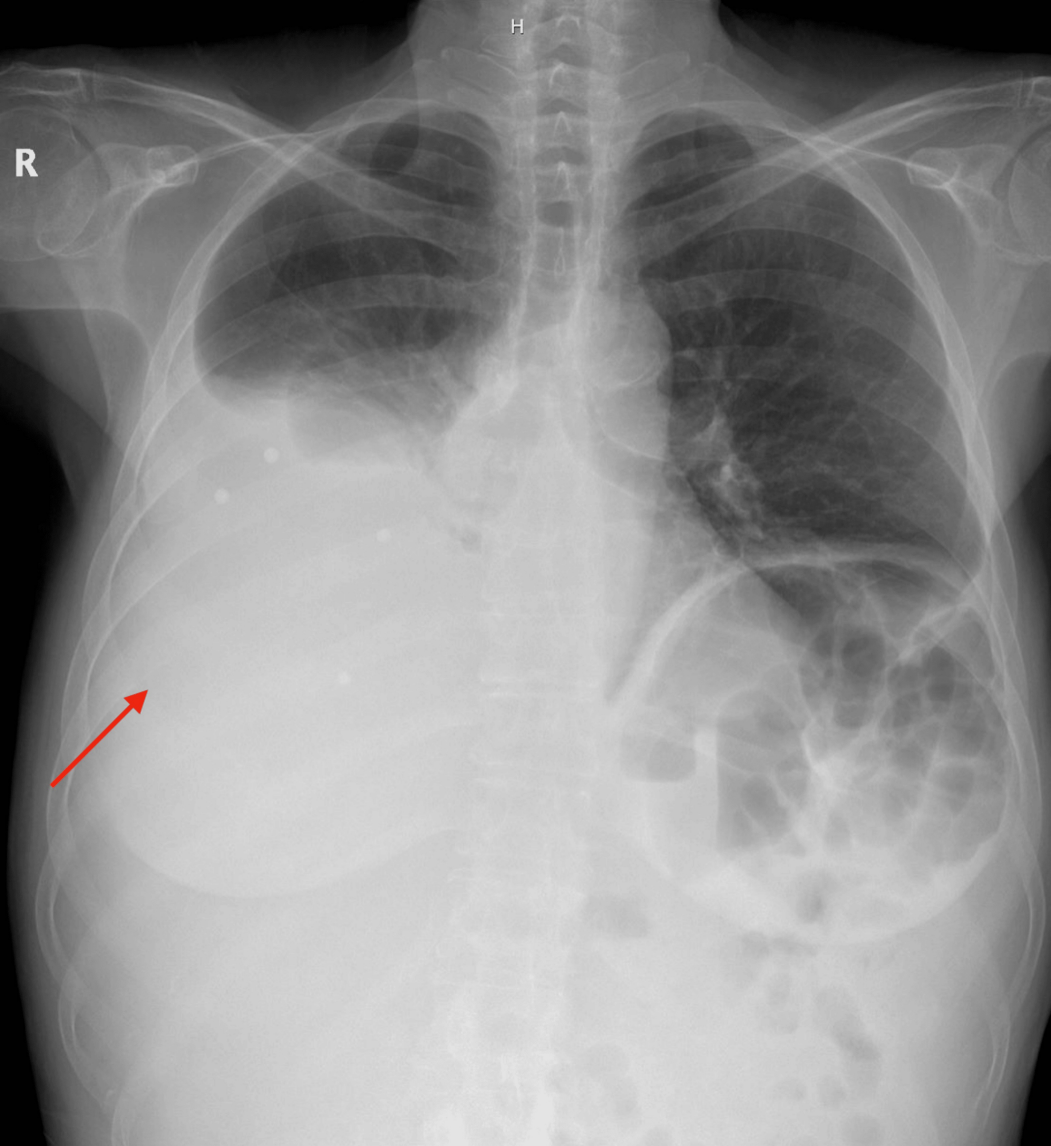
Chest radiograph demonstrating a large right pleural effusion with associated compressive atelectasis. The red arrow highlights the dense opacification and blunting of the right costophrenic angle, consistent with a significant pleural fluid collection.

**Figure 2:**
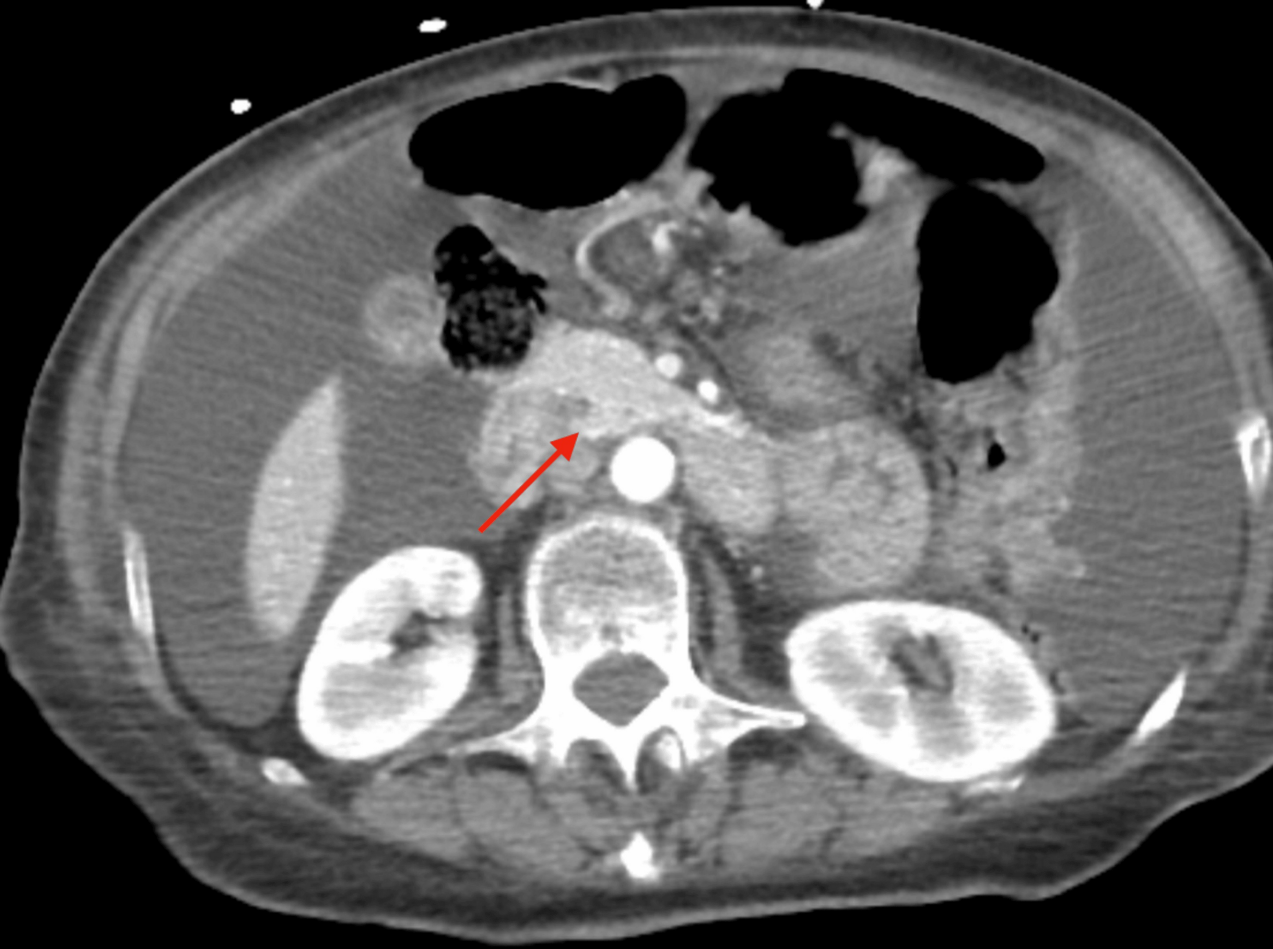
Contrast-enhanced CT scan of the abdomen and pelvis demonstrating diffuse peritoneal thickening and omental caking, characteristic of peritoneal carcinomatosis. The red arrow highlights the region of omental caking, seen as irregular, increased soft-tissue density along the omentum.

A chest X-ray showed a large right pleural effusion (Figure 1). Thoracentesis was performed, yielding 2,000 mL of serous fluid, which improved her respiratory symptoms. Computed tomography (CT) of the chest, abdomen, and pelvis revealed diffuse peritoneal nodularity and omental thickening consistent with peritoneal carcinomatosis (Figure 2). Both ovaries appeared normal, and no adnexal masses were identified. An abdominal ultrasound confirmed the absence of ovarian or uterine lesions.

**Figure 3:**
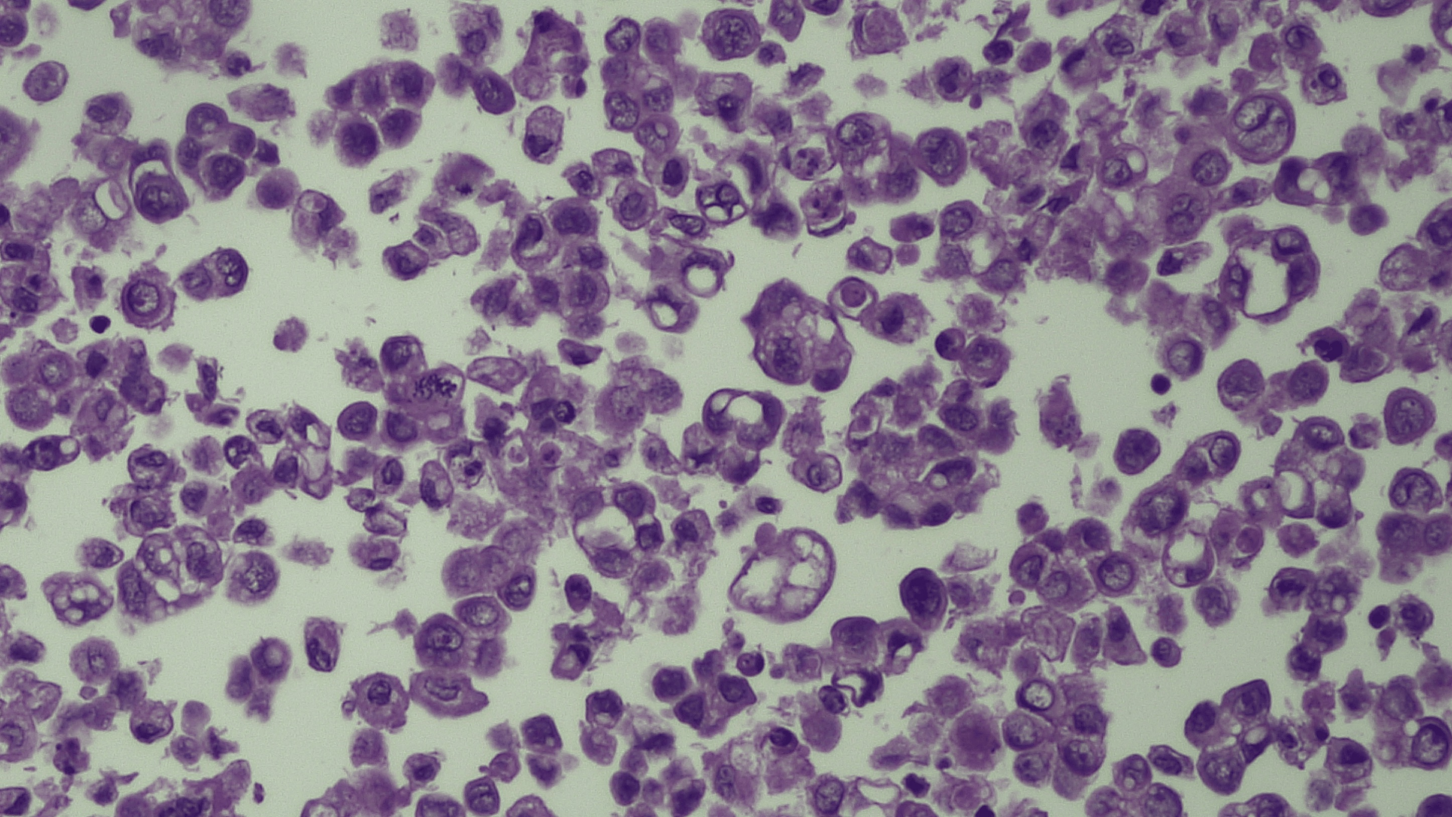
Cytologic examination of ascitic fluid showing numerous clusters and single malignant epithelial cells with enlarged, vesicular nuclei and prominent nucleoli (Hematoxylin & Eosin stain, ×400). Scattered tumor cells exhibit high nuclear-to-cytoplasmic ratios and coarse chromatin, consistent with poorly differentiated adenocarcinoma of Müllerian origin.

Paracentesis was performed for diagnostic evaluation. Cytologic examination of the ascitic fluid demonstrated malignant epithelial cells arranged in clusters and papillary formations, exhibiting enlarged vesicular nuclei with prominent nucleoli and a high nuclear-to-cytoplasmic ratio (Figure 3). These findings were consistent with a poorly differentiated adenocarcinoma of Müllerian origin.

**Figure 4:**
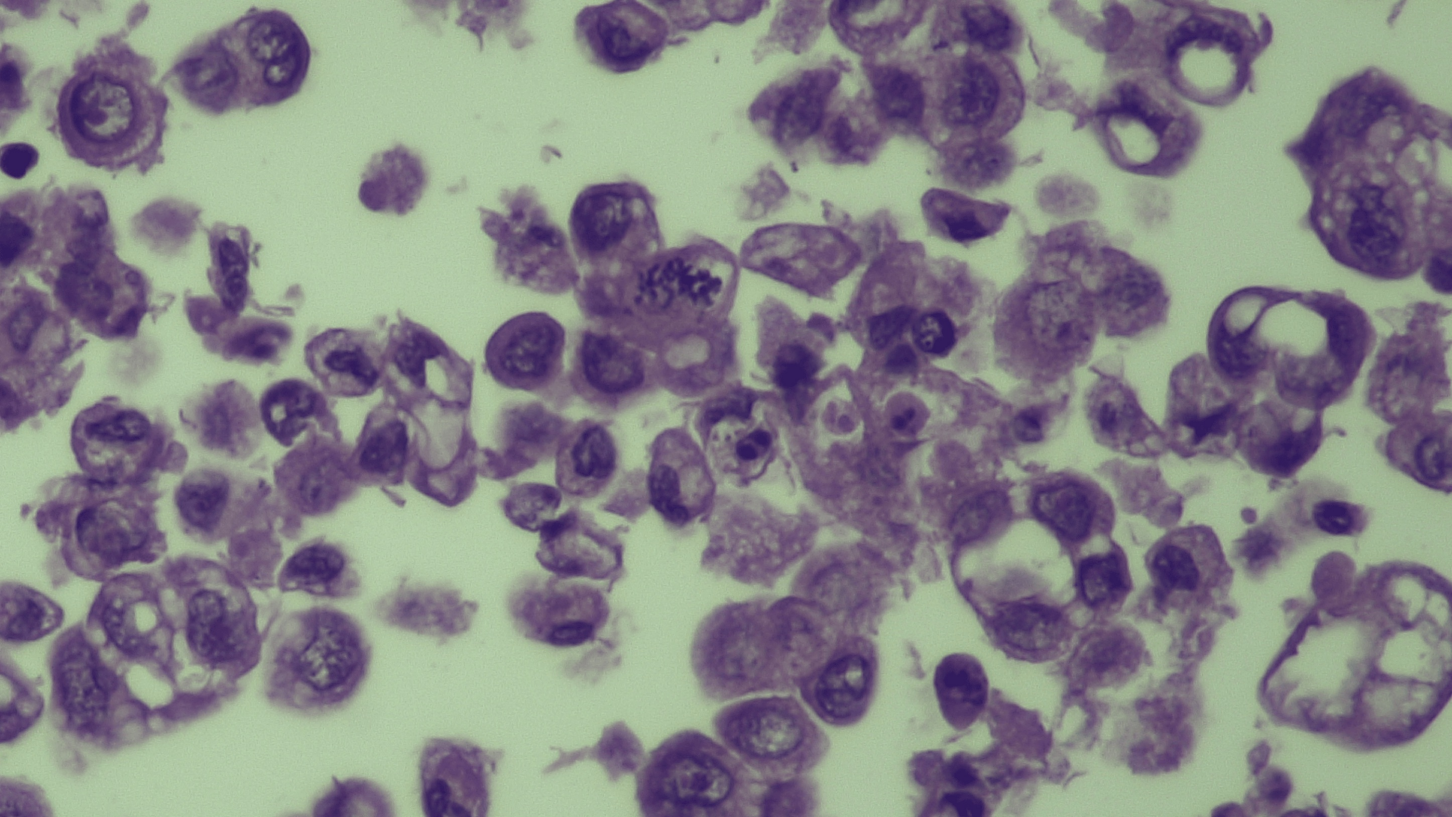
Higher magnification view of the ascitic fluid cytology (H&E stain, ×600) showing pleomorphic neoplastic epithelial cells with irregular nuclei and occasional signet-ring morphology.

A higher magnification view (Figure 4) showed pleomorphic neoplastic epithelial cells with irregular nuclei and occasional signet-ring morphology. Immunohistochemical staining was positive for CK7, PAX8, CK19, and CA-125, and negative for CK20, confirming a Müllerian epithelial origin. PET/CT imaging revealed diffuse peritoneal and omental hypermetabolic activity without any ovarian or gastrointestinal uptake, supporting the diagnosis of primary peritoneal adenocarcinoma.

The patient was evaluated by the oncology team and managed with supportive and palliative care, including serial paracenteses and symptom-directed therapy. She was referred for outpatient oncology follow-up to discuss systemic chemotherapy options.

## Discussion

PPC is a rare malignancy of Müllerian origin that closely resembles EOC in its histologic and immunophenotypic profile, yet it arises from the peritoneal lining rather than the ovaries. The pathogenesis is thought to involve malignant transformation of residual Müllerian epithelium in the peritoneum, which retains the potential for serous differentiation. This embryologic origin explains the near-identical morphology between PPC and high-grade serous ovarian carcinoma [1,2].

Clinically, PPC often presents in postmenopausal women with symptoms of abdominal distension, ascites, or respiratory compromise secondary to pleural effusions, features that were all present in our patient. Radiologic findings commonly include diffuse peritoneal thickening and omental caking without an identifiable ovarian mass, which distinguishes PPC from ovarian primaries [3]. However, imaging alone cannot reliably differentiate between PPC, EOC, and metastatic gastrointestinal adenocarcinomas; thus, cytopathology and immunohistochemistry are essential for definitive diagnosis.

In our patient, the ascitic fluid revealed malignant epithelial cells positive for CK7, PAX8, CK19, and CA-125, and negative for CK20, an immunoprofile consistent with Müllerian serous carcinoma [4]. These markers, along with the absence of ovarian or gastrointestinal lesions on CT and PET/CT, supported the diagnosis of primary peritoneal adenocarcinoma. The immunohistochemical pattern mirrors that observed in other Müllerian tumors, particularly serous ovarian and fallopian tube carcinomas, reinforcing the shared origin of these neoplasms [5,6].

From a diagnostic perspective, PPC remains a diagnosis of exclusion. The Gynecologic Oncology Group (GOG) and the National Comprehensive Cancer Network (NCCN) define PPC as a tumor predominantly involving the peritoneum, with both ovaries normal in size or demonstrating only surface implants less than 5 mm without stromal invasion [7]. These criteria were satisfied in this case, as imaging showed normal-appearing ovaries and diffuse peritoneal involvement.

Therapeutically, PPC is managed similarly to advanced EOC, with cytoreductive surgery followed by platinum-based chemotherapy as the cornerstone of treatment [8]. Optimal debulking has been shown to improve survival outcomes. For patients who are not surgical candidates, as in our case, systemic chemotherapy remains the mainstay of management. The addition of bevacizumab or HIPEC (hyperthermic intraperitoneal chemotherapy) has shown promise in select cases for improving local disease control and progression-free survival [9]. Despite these advances, the overall prognosis remains guarded, with median survival ranging from 12 to 24 months depending on response to therapy [10].

In conclusion, PPC should be suspected in postmenopausal women presenting with peritoneal carcinomatosis and no detectable ovarian mass. Immunohistochemical analysis remains the key diagnostic tool to differentiate PPC from ovarian and metastatic gastrointestinal cancers. Early identification and prompt initiation of platinum-based chemotherapy may improve outcomes. This case highlights the pivotal role of collaborative clinical judgment and diagnostic precision in managing rare Müllerian neoplasms.

## Conclusions

PPC remains a rare but clinically significant malignancy that closely resembles EOC in its presentation and histopathologic features. The absence of an identifiable ovarian mass frequently delays recognition, emphasizing the importance of maintaining a high index of suspicion in patients presenting with ascites and peritoneal carcinomatosis. Accurate diagnosis requires the coordinated integration of imaging, cytopathology, and immunohistochemistry, with Müllerian immunoprofiles, such as CK7, PAX8, CK19, and CA-125 positivity with CK20 negativity, serving as essential diagnostic markers to differentiate PPC from ovarian and gastrointestinal malignancies.

Timely identification of PPC is critical, as prompt initiation of systemic platinum-based chemotherapy and consideration of cytoreductive surgery or HIPEC can improve clinical outcomes. This case underscores the vital role of multidisciplinary collaboration among internists, radiologists, pathologists, and oncologists in ensuring diagnostic precision and optimal patient care. Awareness of PPC and other rare Müllerian neoplasms can prevent misclassification and guide more effective, individualized treatment strategies for patients with unexplained peritoneal carcinomatosis.
